# Supplementary material for: Sustainable Edamame production in an artificial light plant factory with improved yield and quality
Source: Sci Rep. 2025 Sep 12;15:32083. doi: 10.1038/s41598-025-17131-w (PMC12432140; doi:10.1038/s41598-025-17131-w)
Supplement: Supplementary file 1 — Supplementary Material 1 [file 41598_2025_17131_MOESM1_ESM.pdf]

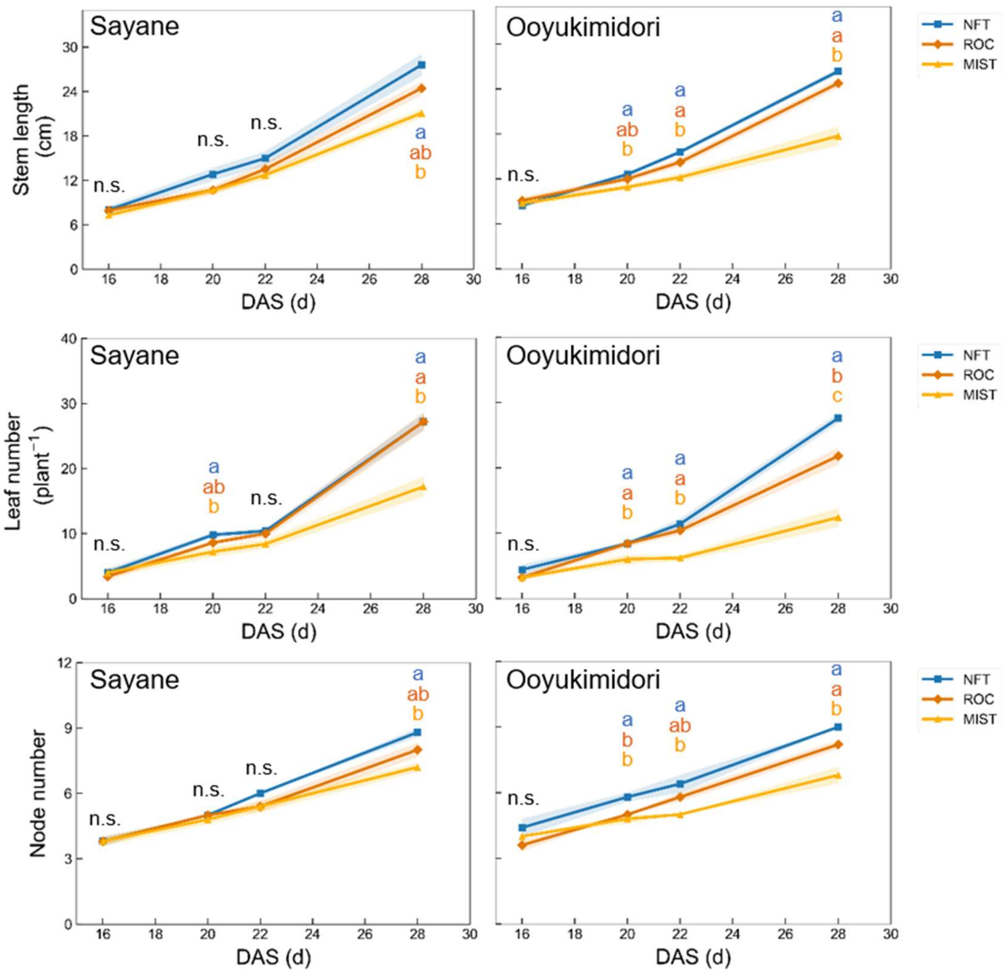

Supplemental Figure S1. Growth traits at vegetative growth stage.

Values are the mean and SE of five replicates. Different letters above the bars indicate significant differences (P<0.05), and n.s. indicates not significant differences (p>0.05) by Tukey's test.

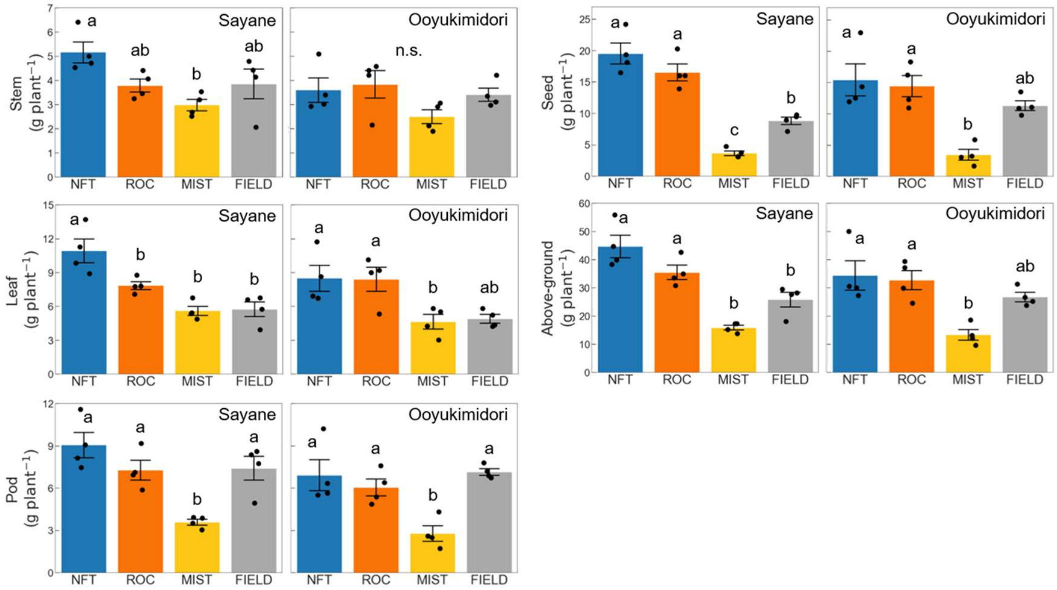

Supplemental Figure S2. Dry matter at maturity.

Above-ground dry weight was calculated as the sum of the dry weights of stem, leaf, pod, and seed. Values are the mean and SE of four replicates. Different letters above the bars indicate significant differences ($P<0.05$), and n.s. indicates not significant differences ($p>0.05$) by Tukey's test.

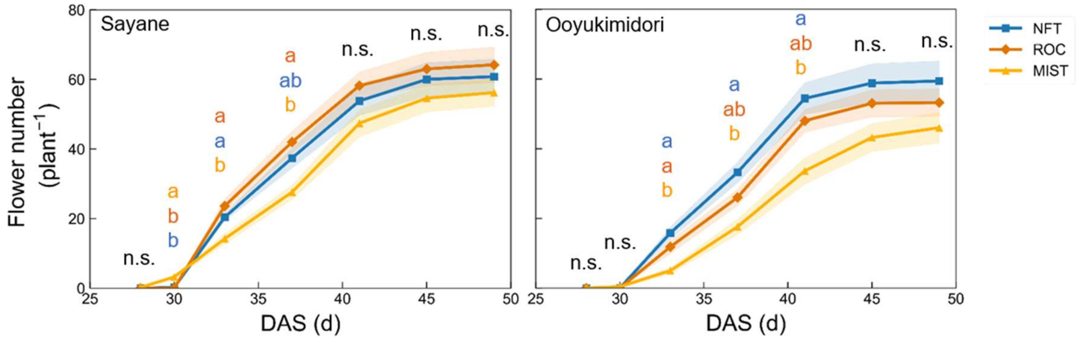

Supplemental Figure S3. Changes in cumulative number of open flowers.

Values are the mean and SE of five replicates. Different letters above the bars indicate significant differences ($P<0.05$), and n.s. indicates not significant differences ($p>0.05$) by Tukey's test.

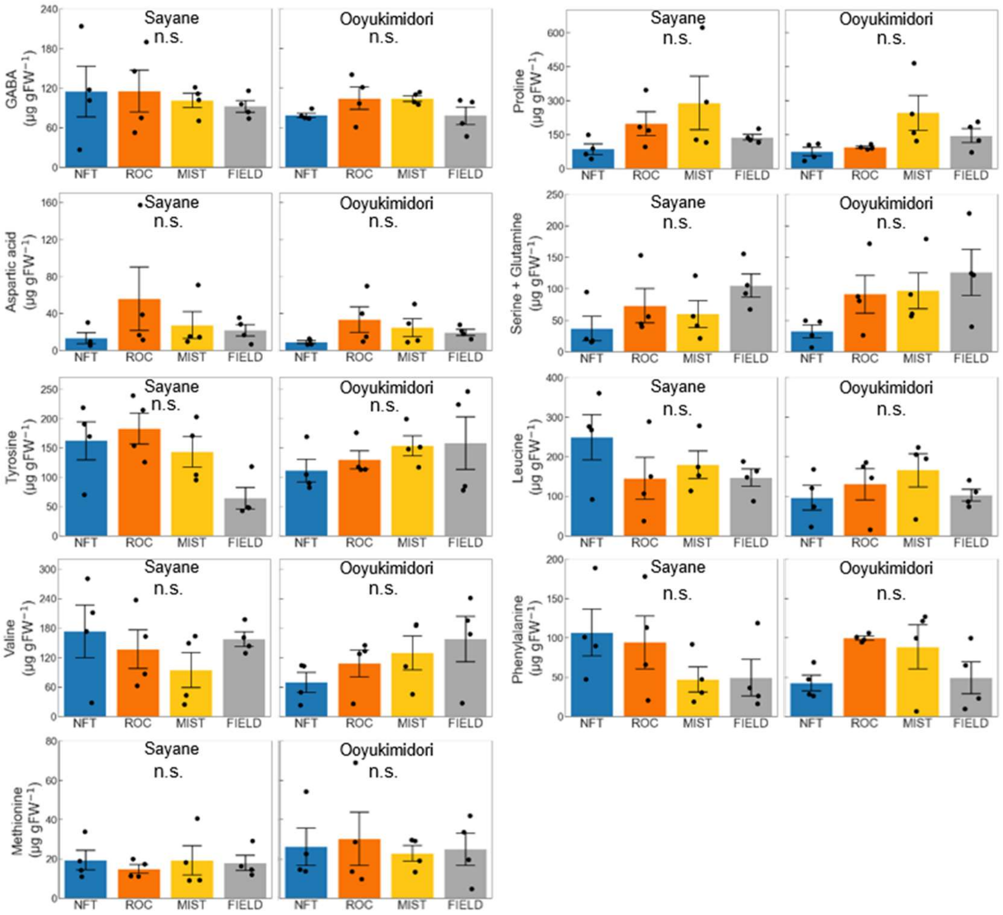

Supplemental Figure S4. Free amino acids content in mature seeds.

Values are the mean and SE of four replicates. Different letters above the bars indicate significant differences ($P<0.05$), and n.s. indicates not significant differences ($p>0.05$) by Tukey's test.

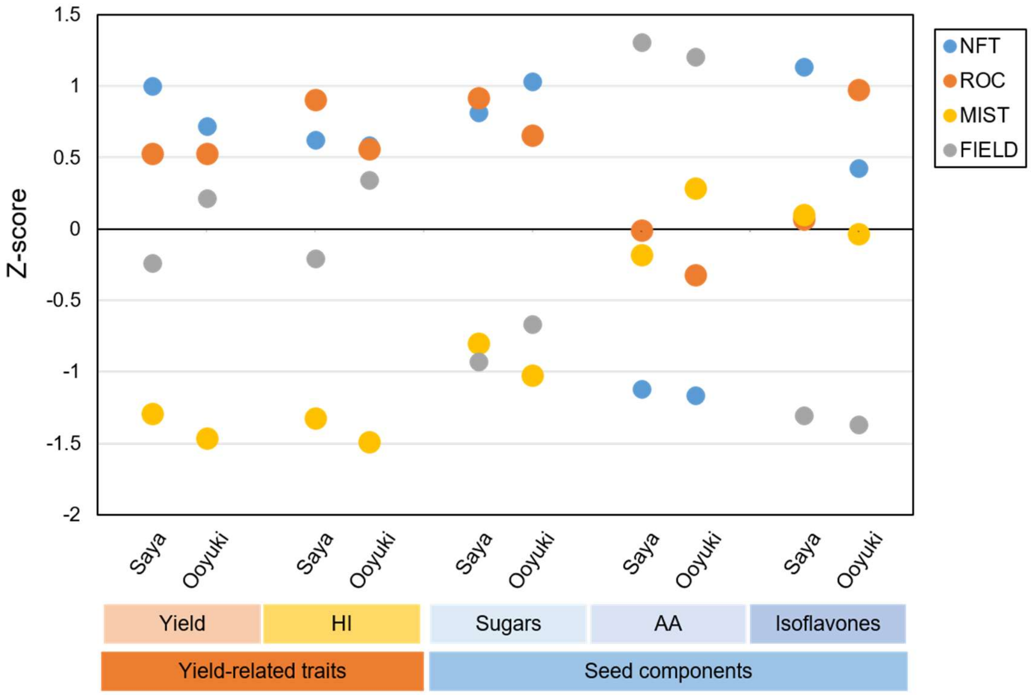

Supplemental Figure S5. Comparison of performance among cultivars in each cultivation system. AA: total free amino acids content, HI: harvest index, Isoflavones: total isoflavones contents, Sugars: total soluble sugar content, Yield: seed fresh weight per plant. Values indicate standardized z-scores.

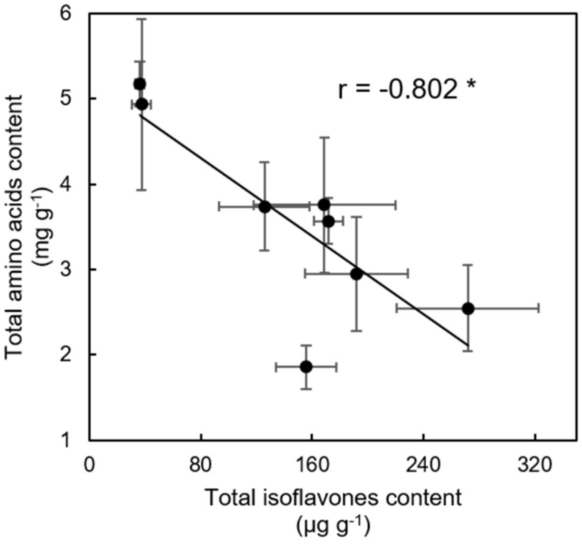

Supplemental Figure S6. Relationship of total isoflavones content with total amino acids content.

Values are the mean and SE of four replicates. * indicates significant differences at the 0.05 level.

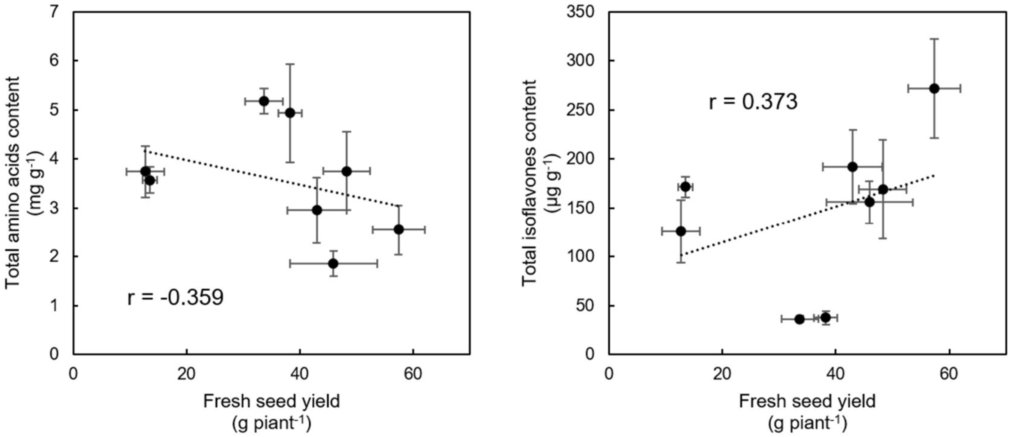

Supplemental Figure S7. Relationships of the fresh seed yield with total amino acids and total. Values are the mean and SE of four replicates.

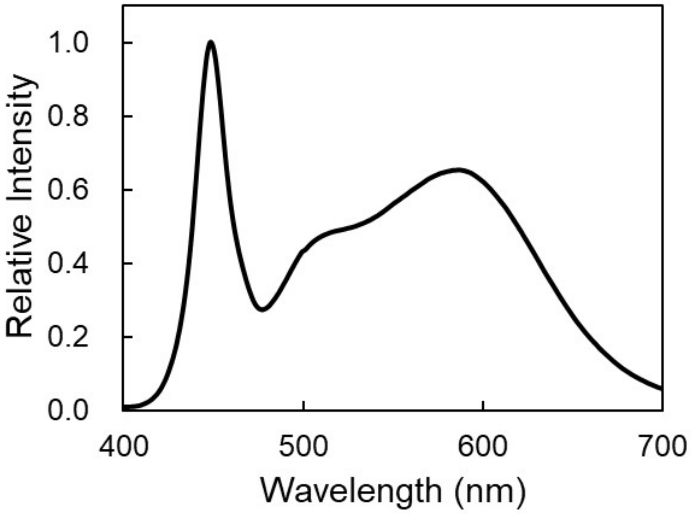

Supplemental Figure S8. Light spectrum of the LEDs used for plant cultivation in this study.

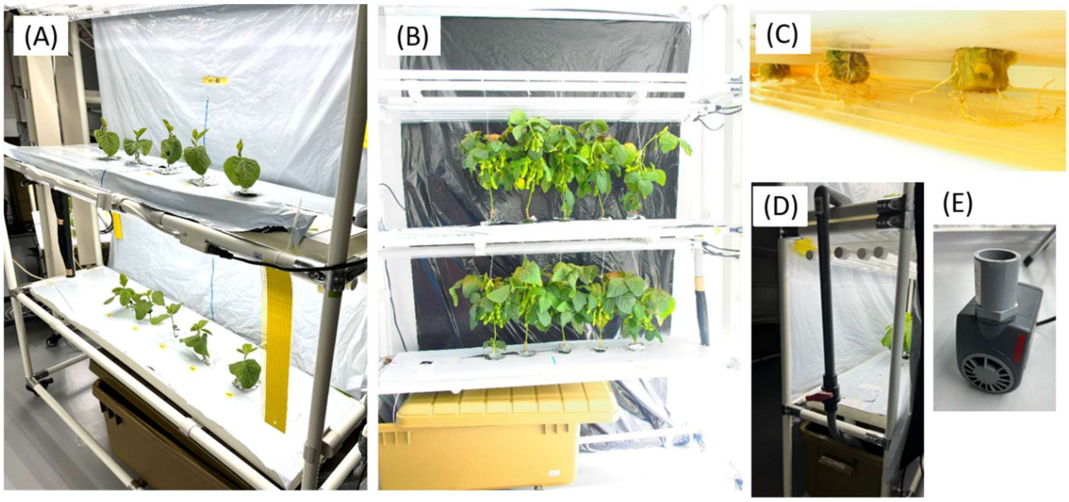

Supplemental Figure S9. Equipment in nutrient film technique (NFT).

(A, B) Cultivation appearance in NFT, (C) Supply of culture medium to root zone, (D) Circulation system of culture medium, (E) A pump used to circulate culture medium.

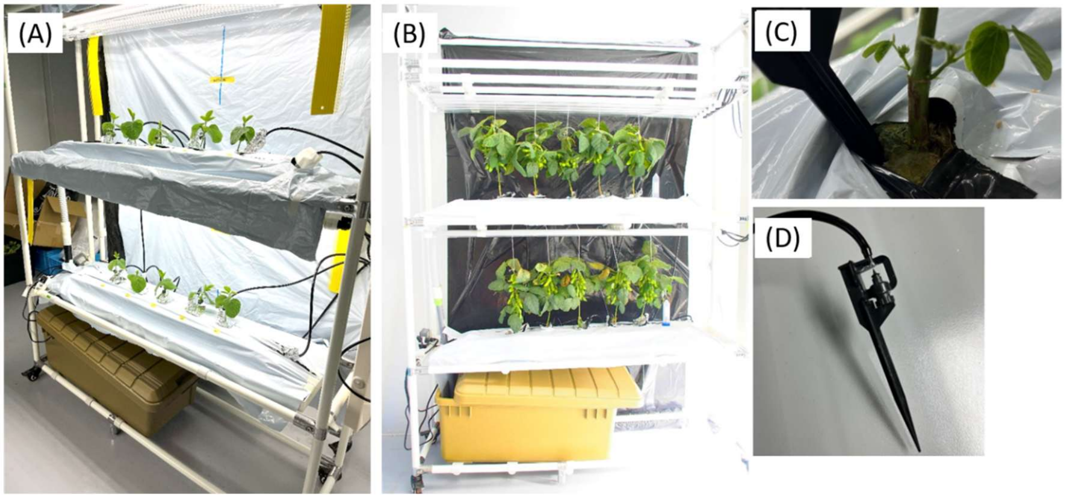

Supplemental Figure S10. Equipment in rockwool culture (ROC).

(A, B) Cultivation appearance in ROC, (C) Drip irrigation at the base of plant using a sprinkler, (D) A plug-in sprinkler used in ROC.

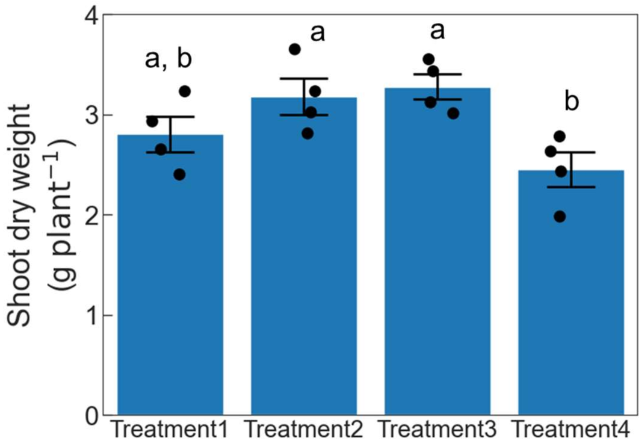

Supplemental Figure S11. Effects of nutrient solution application frequency on plant growth in rockwool. Four irrigation treatments were tested using the edamame cultivar Sayane: (1) nutrient solution applied once every 12 hours for 5 minutes; (2) twice every 12 hours for 2.5 minutes each time; (3) once every 24 hours for 5 minutes; and (4) once every 48 hours for 5 minutes. Shoot dry weight was measured at 30 days after sowing (DAS). Values represent the mean ± SE of four replicates. Different letters above the bars indicate significant differences ($P < 0.05$) according to Tukey's test. In treatment (1), excessive accumulation of nutrient solution in the tray likely led to waterlogging conditions, resulting in impaired plant growth. In contrast, treatments (2) and (3) provided an appropriate water supply, supporting optimal shoot biomass accumulation. In treatment (4), the rockwool substrate dried out before the next irrigation cycle, which likely caused water stress and suppressed plant growth.

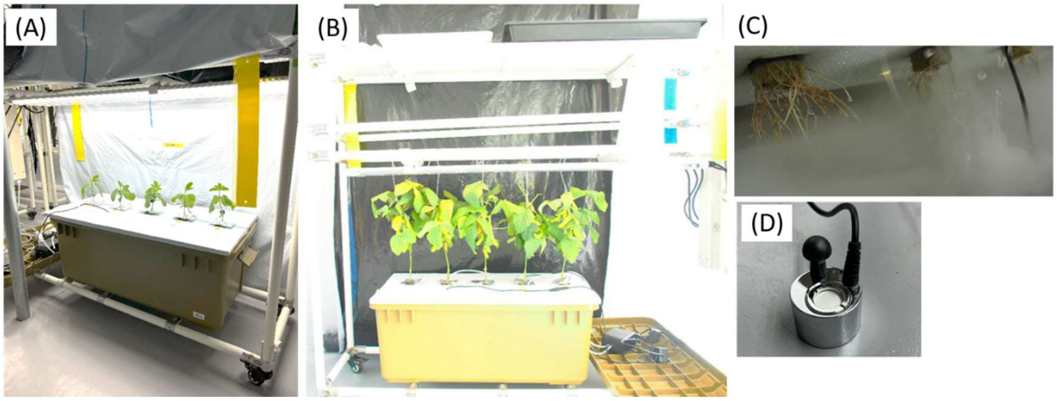

Supplemental Figure S12. Equipment in mist culture (MIST).

(A, B) Cultivation appearance in MIST, (C) Supply of culture medium to root zone by mist filling

(D) An ultrasonic mist generator used in MIST.

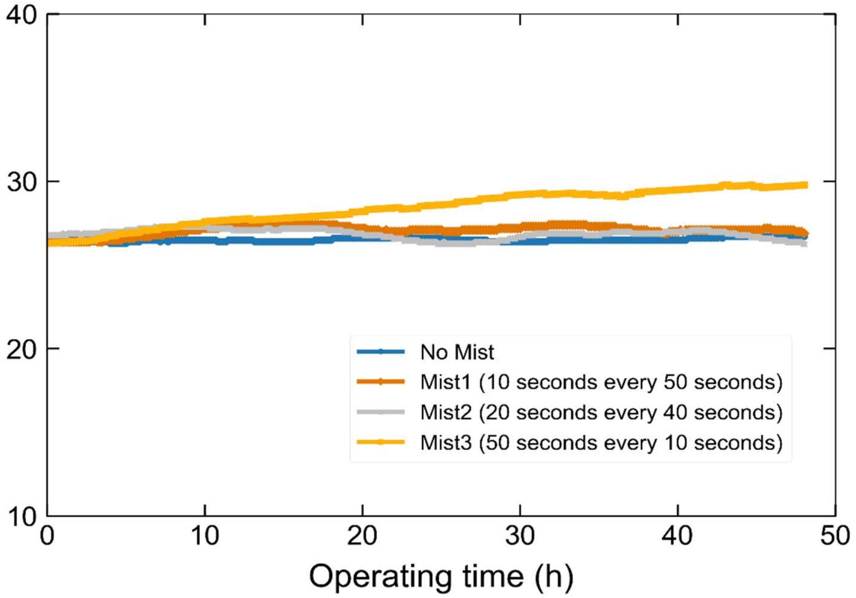

Supplemental Figure S13. Effects of mist generator operating time on nutrient solution temperature in a mist cultivation system. An ultrasonic mist generator (Mini-Mist Maker HS0276, AGPTTEK) was placed inside the container to generate mist. Four treatments were compared: (1) No Mist; (2) Mist 1 (10 seconds ON / 50 seconds OFF); (3) Mist 2 (20 seconds ON / 40 seconds OFF); and (4) Mist 3 (50 seconds ON / 10 seconds OFF). In Mist 3, the nutrient solution temperature increased markedly due to the high duty cycle. Therefore, Mist 2 was selected as the optimal condition that allows high-frequency misting while minimizing the rise in nutrient solution temperature.

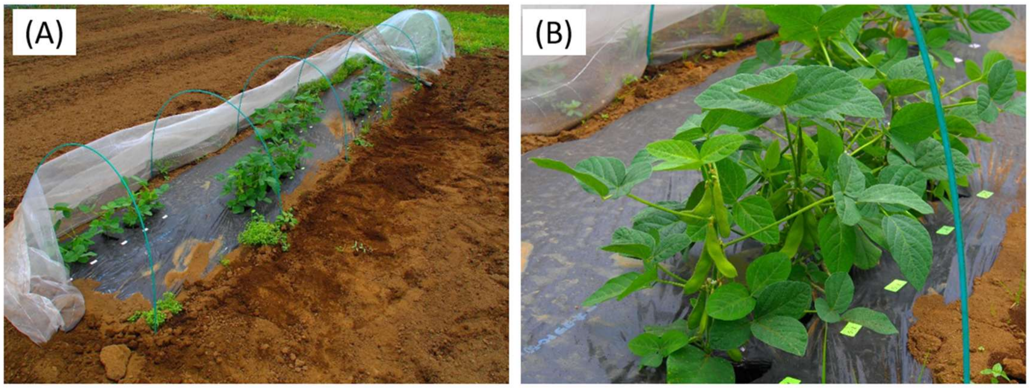

Supplemental Figure S14. Plant cultivation in the field (FIELD).

(A, B) Field cultivation.

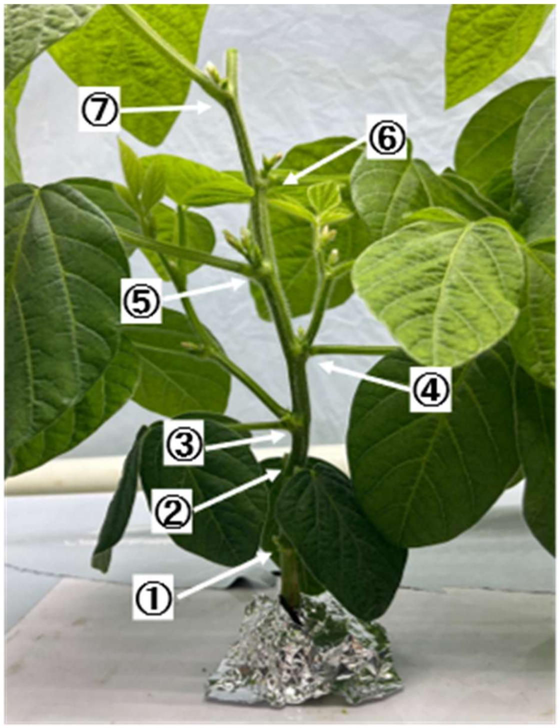

Supplemental Figure S15. Pinching treatment.

Pinching treatment was conducted by cutting off the growth point, the upper 10mm of the seventh node, with scissors at the time the seventh node was visible. In fact, this treatment was conducted as needed after 28DAS.

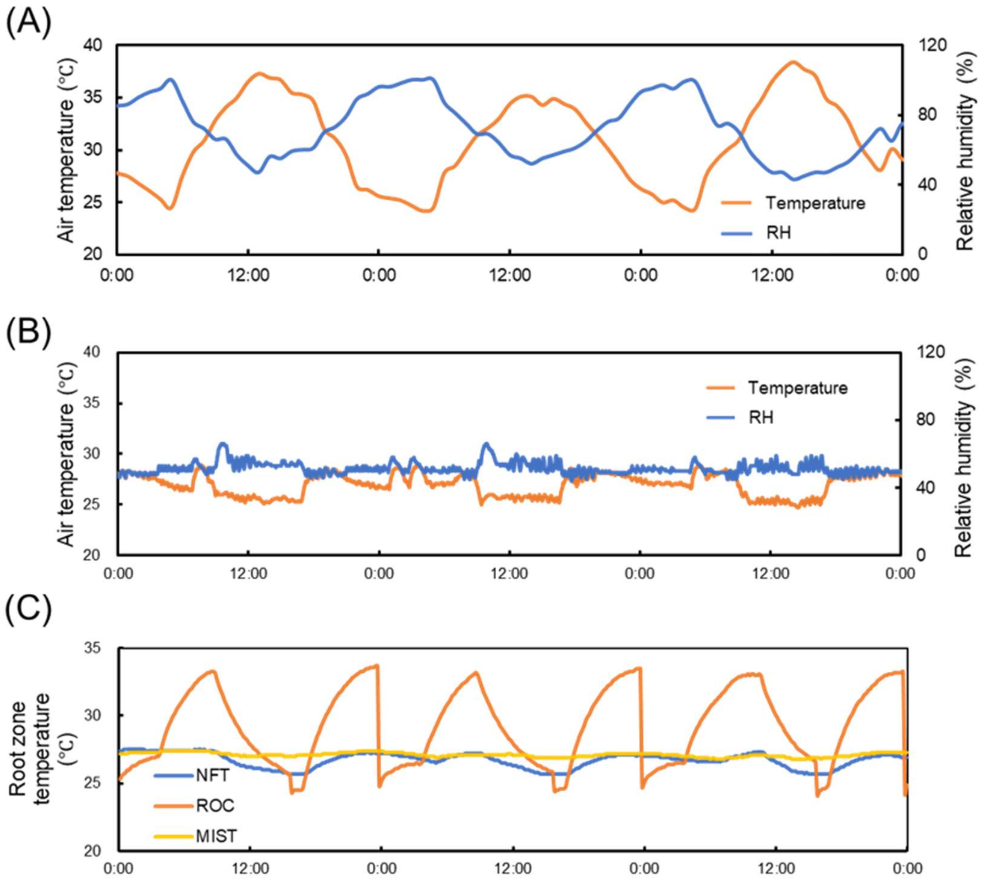

Supplemental Figure S16. Environmental condition from July 9, 2023 to July 12, 2023 in plant factory and field. (A) Air temperature and relative humidity in field, (B) Air temperature and relative humidity in plant factory, (C) Root zone temperature in each cultivation system of plant factory.

Supplemental Table S1. Analysis of variance (ANOVA) for the contents of isoflavones and free amino acids.

		Isoflavones					Free amino acids				
Cultivar (C)	System (S)	Malonyl genistin ($\mu g\ gFW^{-1}$)	Malonyl daidzin ($\mu g\ gFW^{-1}$)	Daidzin ($\mu g\ gFW^{-1}$)	Glycitein ($\mu g\ gFW^{-1}$)	Total isoflavones ($\mu g\ gFW^{-1}$)	Asparagine ($\mu g\ gFW^{-1}$)	Alanine ($\mu g\ gFW^{-1}$)	Glutamic acid ($\mu g\ gFW^{-1}$)	Arginine ($\mu g\ gFW^{-1}$)	Total amino acids ($mg\ gFW^{-1}$)
Sayane	NFT	119.5 a	77.5 a	61.8 a	12.9 a	271.7 a	201 b	538 ab	24 a	819 a	2.55 ab
	ROC	66.9 abc	59.4 ab	35.4 ab	7.1 ab	168.9 ab	813 ab	812 ab	91 a	1020 a	3.75 ab
	MIST	74.1 abc	56.0 ab	35.4 ab	5.9 ab	171.4 ab	595 ab	757 ab	122 a	1130 a	3.57 ab
	FIELD	30.0 c	5.8 b	0.4 b	0.1 b	36.2 b	1469 a	1306 a	299 a	1305 a	5.17 a
Ooyukimidori	NFT	49.8 bc	81.8 a	19.9 b	4.4 ab	155.9 ab	306 b	116 b	33 a	861 a	1.86 b
	ROC	104.6 ab	52.1 ab	28.5 ab	6.6 ab	191.9 a	783 ab	348 ab	84 a	914 a	2.95 ab
	MIST	60.1 bc	44.5 ab	16.8 b	4.3 ab	125.7 ab	957 ab	706 ab	268 a	772 a	3.74 ab
	FIELD	22.1 c	10.6 b	2.3 b	2.6 ab	37.6 b	1288 a	1375 a	213 a	1195 a	4.93 a
mean	Sayane	72.6 a	49.7 a	33.2 a	6.5 a	162.1 a	770 a	853 a	134 a	1069 a	3.76 a
	Ooyukimidori	59.2 a	47.2 a	16.8 b	4.5 a	127.7 a	834 a	636 a	149 a	935 a	3.37 a
mean	NFT	84.7 a	79.6 a	40.8 a	8.6 a	213.8 a	254 b	327 b	29 b	840 a	2.20 b
	ROC	85.8 a	55.8 a	32.0 a	6.9 ab	180.4 a	798 b	580 b	87 ab	967 a	3.35 b
	MIST	67.1 a	50.3 a	26.1 a	5.1 ab	148.6 a	776 b	731 ab	195 ab	951 a	3.65 ab
	FIELD	26.1 b	8.2 b	1.3 b	1.3 b	36.9 b	1379 a	1340 ab	256 a	1250 a	5.05 a
ANOVA	Cultivar (C)	n.s.	n.s.	**	n.s.	n.s.	n.s.	n.s.	n.s.	n.s.	n.s.
	System (S)	***	***	***	*	***	***	**	**	n.s.	***
	C×S	**	n.s.	n.s.	n.s.	n.s.	n.s.	n.s.	n.s.	n.s.	n.s.

Values are the mean and SE of four replicates. Different letters indicate significant differences at the 0.05 level. ** and *** indicate significant differences at the 0.01 and 0.001 levels, respectively, and n.s. indicates not significant differences.
